# Excessive Weight Gain and Dental Caries Experience among Children Affected by ADHD

**DOI:** 10.3390/ijerph17165870

**Published:** 2020-08-13

**Authors:** Elzbieta Paszynska, Monika Dmitrzak-Węglarz, Aleksandra Perczak, Maria Gawriolek, Tomasz Hanć, Ewa Bryl, Paula Mamrot, Agata Dutkiewicz, Magdalena Roszak, Marta Tyszkiewicz-Nwafor, Agnieszka Slopien

**Affiliations:** 1 Department of Integrated Dentistry, Poznan University of Medical Sciences, Poznan 60-812, Poland; euro-dent@wp.pl (A.P.); mgawriolek@gmail.com (M.G.); 2 Psychiatric Genetics Unit, Department of Psychiatry, Poznan University of Medical Sciences, Poznan 60-806, Poland; mweglarz@ump.edu.pl; 3 Institute of Human Biology and Evolution, Faculty of Biology, Adam Mickiewicz University, Poznan 61-614, Poland; tomekhanc@gmail.com (T.H.); ewabryl2@gmail.com (E.B.); mamrotpaula@gmail.com (P.M.); 4 Department of Child and Adolescent Psychiatry, Poznan University of Medical Sciences, Poznan 60-572, Poland; dutkiewicz.agata22@gmail.com (A.D.); malamt@gmail.com (M.T.-N.); asrs@wp.pl (A.S.); 5 Department of Computer Science and Statistics, Poznan University of Medical Sciences, Poznan 60-806, Poland; mmr@ump.edu.pl

**Keywords:** caries, ADHD, overweight, sugar intake, ICDAS

## Abstract

In recent years, attention has been paid to the co-occurrence of attention deficit hyperactivity disorder (ADHD) and obesity, but results in relation to dental caries outcomes differ. The study was conducted to determine obesity/overweight and dental caries in children suffering from ADHD and to draw comparisons with non-ADHD children. A total of 119 children under 11 years old (8.2 ± 1.2) were enrolled into a cross-sectional study: those with confirmed ADHD (*n* = 39), and healthy controls (*n* = 80). The behavioral evaluation included a parent interview directed at sweetened food/drink habits. The clinical evaluation included physical measurements (height, waist, hip circumference, body weight, body mass index (BMI), and dental examination (International Caries Detection and Assessment System—ICDAS). Results showed a higher prevalence of abnormal body weight, hip circumference, and BMI, and a higher frequency of caries (84.6%) in the ADHD group. Significant caries differences for primary (ICDAS 0, 1, 2, 5, 6 scores) and permanent teeth (ICDAS 1, 3 scores) were recorded. The questionnaire pinpointed interplays between sugar consumption and tooth decay, especially for primary dentition. It can be concluded that the consumption of sweetened foods/drinks among ADHD children may lead to an increased rate of overweight, but may also affect oral health. Limiting sugar consumption might be one of the important elements in prevention programmes against dental caries and overweight/obesity.

## 1. Introduction

Attention deficit hyperactivity disorder (ADHD) is one of the most commonly diagnosed neurodevelopmental disorders among school children. Its characteristics are permanent behavioral patterns such as motoric hyperactivity, impulsiveness, and attention deficit [1]. Depending on the diagnostic criteria and population, the prevalence of ADHD among children is from 1–2% (according to the International Statistical Classification of Diseases and Related Health Problems (10th ed.), ICD-10) [2] to 3.5% (according to the Diagnostic and Statistical Manual of Mental Disorders (5th ed.), DSM-V) [3], with the average global prevalence in children and adolescents equaling 5.3% [4,5]. In recent years, more attention has been paid to the co-occurrence of ADHD and obesity or excess of weight [6,7,8,9,10,11]. Most studies show that ADHD is a risk factor for obesity independently of pharmacological treatment, socio-economic status, and most of the other disorders accompanying ADHD. The link between ADHD and obesity becomes more pronounced with age, from a slight correlation in toddlers with ADHD to a high frequency of obesity in adults with ADHD [12,13]. However, in relation to the general population, international studies have reported that the number of persons with overweight and obesity almost tripled in the last 40 years [14]. The World Health Organization (WHO) indicated that the global prevalence of overweight or obesity among children and adolescents aged 5–19 had increased from 4% in 1975 to over 18% in 2016 [15]. In Poland, a similar trend was recorded among youth who were overweight or obese, from 17.8% to 21.9%, respectively [16]. A recent study by Dmitrzak-Weglarz et al. reported that 78.5% of Polish school children had declared that they reached for snacks between meals [17]. This is consistent with previous studies that sweet snacks between meals were declared by 90% of children [18].

Besides, earlier studies have shown that ADHD is associated with dysfunctional eating patterns such as skipping breakfast, eating in the evening and at night, eating and drinking more during the day, impulsive and emotional eating, preferences for high caloric food, eating more snacks and junk food in comparison to peers, increased focus on eating, and significantly more bulimic behaviors [1]. There are still several explanations for the mechanisms responsible for excessive food consumption; hyperactivity and impulsivity are proposed to be linked with ADHD [19]. The impact of the abovementioned inadequate eating habits can also explain the broader common determinants of overweight and obesity in ADHD.

It can be assumed that some intermediary factors coexist, and one of them is sugar consumption [20,21,22,23]. Unhealthy dietary habits related to ADHD may lead to increased rate of overweight in this group of patients, but may also affect oral health. The relationship between children with ADHD and caries has been taken into account, along with the tendency to put on weight, even though oral health is only one essential aspect of child development. In recent years, attention has been paid to the co-occurrence of ADHD and obesity, but results concerning dental caries outcomes differ. There are conflicting results in scientific reports according to dental status and significant obesity predictors [24,25,26,27,28]. Besides, these surveys of ADHD and caries are due to definitive dentition, and information concerning primary dentition is limited due to the subjects being aged over 12 years [24,25,26,27,28].

ADHD may be associated with poor oral hygiene. This connection was analyzed in clinical and behavioral studies including parent interviews by Chandra et al., where dental caries prevalence in the primary dentition was increased in the children suffering from ADHD due to poor oral hygiene and increased consumption of sugary foods [29]. Another study concluded that at the age of 13, children with ADHD were not exposed to statistically significant higher tooth decay, but still had poorer oral hygiene due to poor behavioral habits compared to the control group [25]. Conclusions from the abovementioned studies suggest that children with ADHD need more support from their parents in terms of diet and hygiene. Our recent study intended to explore a part of these determinants that were previously investigated in the other reports. The purpose of the cross-sectional study was to determine the obesity/overweight and dental caries experiences of children suffering from ADHD and compare them with those of non-ADHD children. This study tested the hypothesis that children with ADHD exhibit a higher prevalence of overweight/obesity and caries than healthy children. Therefore, examined the use of anthropometric indicators of excessive weight gain and the prevalence of dental caries, along with dietary habits such as sugar consumption.

## 2. Materials and Methods

### 2.1. Sample of the ADHD Group

We planned an enrollment of children with ADHD from the Poznan region, living permanently in this part of the country (western part of Poland). The announcement about the recruitment for children with suspected ADHD was forwarded to the family doctors’ outpatient clinics (*n* = 100) and mental health outpatient clinics for children and youth (*n* = 20). Sixty-six parents of children with unconfirmed ADHD diagnosis responded to the announcement, agreeing to include their child in the investigation. Finally, in sixty-six children the diagnosis was verified independently by both a psychiatrist and clinical psychologist, in accordance with ICD-10 and DSM-V diagnostic criteria [2,3]. Among sixty-six children reported to the medical examination, the diagnosis was confirmed in fifty-four subjects. Fourteen children were excluded due to a lack of cooperation during dental examination in the dental office, and the parents of one child withdrew their consent. Finally, the study group consisted of thirty-nine children with confirmed ADHD diagnosis, with ages ranging from 6 to 11 years (8.8 ± 1.2; 31 boys (79.5% all participants in the study group) and 8 girls (20.5%, respectively)).

### 2.2. Sample of the Control Group

Four primary schools were randomly selected from the northern, southern, eastern, and western parts of the Poznan agglomeration. Project representatives directly proposed that all parents of children aged from 6 to 12 years old assess their child’s biological development and oral health in a dental office (free dental consultation in one dental office). Parents registered four hundred children for participation in the project; one hundred and fifty children were selected for the control group according to the inclusion criteria (Table 1). After the general interview, fifty children did not meet inclusion criteria according to age (over 12 years old) or were absent from school for longer than one month; an additional twenty children refused cooperation directly during a dental examination. Finally, eighty children were allocated into the control group, with ages ranging 6–10 years (8.0 ± 1.1; 41 boys (51.3% of the control group) and 39 girls (48.7%, respectively)), recruited among primary schoolchildren.

### 2.3. Ethical Approval

The methodology of this cross-sectional research assumed non-invasive methods of analysis such as the body measurement of the children, dental examination, and questionnaire provided to the children taking part in the project. The protocol of the study followed the standards of Good Clinical Practice (GCP) and was accepted by the local Ethics Committee at the University of Medical Sciences (resolution no. 26/18) [31]. All children participating in the project have been informed of the aims and types of research, and their legal guardians have submitted written consent.

### 2.4. Assessment of Child Development

The clinical evaluation of health status included general body measurements such as height, waist, hip circumference, and body weight. Body height in a standing position was measured with the SECA 216 (SECA, Hamburg, Germany) wall-mounted stadiometer with an accuracy of 0.1 cm. Body weight was recorded in lightweight clothing on a digital scale with an accuracy of 0.1 kg. The waist circumference was determined halfway between the lower edge of the rib arch and the upper iliac crest using metric tape [32]. The hip circumference was measured at a level parallel to the floor, at the largest circumference of the buttocks using metric tape [32]. All subjects were assessed for body mass index (BMI) according to the formula [33]

(1) $$\mathrm{BMI}=\frac{\mathrm{weight}\;\left(\mathrm{kg}\right)}{{\left(\mathrm{height}\right)}^{2}\left(m^{2}\right)}$$

### 2.5. Interview and Oral Examination

In both groups the researcher (A.P.) filled in a questionnaire based on the individual conversation with parents and children; an interview included questions about the history of dental care as well as dietary habits, with an emphasis on the consumption of fermentable carbohydrates in food and beverages. An oral examination was performed with the use of sterile dental diagnostic sets in artificial light in accordance with the aseptic rules performed in the dental office. The sets consisted of a dental mirror, dental probe, sterile pads, and an air-water spray tip. During the examination, rubber gloves and protective masks were used. The methodology of the study included the dental examination of teeth with the criteria of the International Caries Detection and Assessment System (ICDAS II) [34]. For the ICDAS, the detection of caries was performed by recording the two-digit codes for each tooth surface: for tooth surface classification, choosing among sound, sealed, restored, crowned, or missing, and the latter, for the caries stage assessment. The results were transferred onto the original medical chart. The ICDAS is a clinical point system allowing for the detection and classification of caries lesions and estimating their activity level. The ICDAS was developed for clinical purposes to control the progress or regression of the caries disease and evaluate preventative activity. The system can be used to assess the surface of crowns and roots of the teeth, and in contrast to other systems it classifies dental lesions in the enamel, which are not cavities. ICDAS assesses the altered surfaces and estimates the potential depth of the caries changes on a scale from 1 to 6, where 0 means a healthy, unchanged enamel [34,35].

Dental and anthropometric examiners were thoroughly experienced researchers who had led similar subject measurements in several clinical studies [9,17,36]. The dentist (E.P.) involved in the clinical oral assessment had a previous course for standard evaluation and was trained before the onset of the survey by using the practical training under the guidance of an experienced cariologist. The skills of the investigator were taught at the start of the study and retrained twice more during the course of the study using a training tool especially developed for this study (assessment of almost thirty clinical photographs of teeth with different scores, in a random sequence). To test the interexaminer reliability, linear and squared weighted Kappa coefficients were calculated.

The parents’ questionnaire was designed as a question/answer sheet and individual filling. The examiner involved in the anthropometric assessment was always the same person which administered the questionnaire.

### 2.6. Statistical Analysis

The analyzed data are expressed as mean ± standard deviation, median, minimum and maximum values, interquartile range, or percentage, as appropriate. Normality of distribution was tested using the Shapiro–Wilk test, and equality of variances was checked using Levene’s test. Comparison of two unpaired groups was performed using either the unpaired *t*-test for data that followed a normal distribution and had homogeneity of variances or the Mann–Whitney U-test. The relationship between variables was analyzed with Spearman’s rank correlation coefficient and categorical data were analyzed with the χ^2^ test. All results were considered significant at *p* < 0.05. Statistical analyses were performed with STATISTICA 13.0 (StatSoft Inc, Tulsa, OK, USA).

## 3. Results

### 3.1. Assessment of Physical Development among Children

Assessment of the present physical development of children was carried out based on body measurements and showed significant differences in parameters such as body weight, hip circumference, and BMI (respectively *p* = 0.008, *p* = 0.001, *p* = 0.016) between the sample and control groups. Other parameters like height and waist circumference were not significantly different between groups (respectively *p* = 0.103, *p* = 0.365), as can be seen in Table 2.

### 3.2. Results of Dental Examination with ICDAS Score

The use of the ICDAS II index (0–6 degrees) for primary and permanent teeth was performed for all participants. In the ADHD group, the condition of primary dentition was definitely worse in comparison with the control group, with the frequency of caries of 84.6% (41.6% in the control group). ICDAS II showed significant differences for ICDAS codes 0, 1, 2, 5, 6 with unfavorable results for children with ADHD, who compared to healthy children had more primary teeth with advanced caries lesions (Table 3).

The condition of permanent teeth was also statistically different between groups, concerning the number of teeth with caries. Based on the ICDAS score, the results showed significant differences in ICDAS codes 1 and 3, with unfavorable outcomes for children with ADHD (*p* = 0.071, *p* = 0.01 respectively) who compared to healthy children had more permanent teeth with advanced caries lesions (Table 4).

### 3.3. Results of the Interview According to Sweetened Food and Drink Habits

The results of parent interviews indicate that children with ADHD frequently eat sweetened food products containing fermentable carbohydrates in the form of snacks between main meals (*p* < 0.001), and supervision from the parents is limited (*p* < 0.0005).

In the ADHD group a number of positive answers on sweet snack/drink-related questions differed from the control group. The consumption of carbonated or sweetened beverages was declared to be more frequent in the ADHD group than among the control group (*p* < 0.001, *p* < 0.0004 respectively), as can be seen in Table 5.

### 3.4. Relationships between Sweetened Snacks, Dental Caries, and Physical Development

In the ADHD group, statistical analysis (Mann–Whitney U-Test) evidenced a positive relationship between declared sweetened snacks consumption and ICDAS 1, 3 scores in primary teeth (*p* < 0.0003, *p* < 0.02 respectively), as well as eating without supervision and ICDAS code 3 in primary teeth (*p* < 0.02). There were no statistical correlations between all declared nutritional habits and ICDAS results in permanent teeth. Body measurement calculations were not significantly related to sweetened food/drink consumption (*p* > 0.05). Nevertheless, it is worth noting that the relationship between waist circumference and the drinking of carbonated sweet drinks was nearly statistically significant in the ADHD group (*p* = 0.086). There was also an important negative relationship revealed between healthy permanent teeth (ICDAS 0 score) and BMI (r = −0.32; *p* < 0.04).

## 4. Discussion

In this study, the biological assessment of children with ADHD included anthropometric measurements such as height, waist and hip circumference, body weight, and BMI. Our results show a significant increase in body weight, hip circumference, and BMI. Height and waist circumference did not show significant differences.

Other studies published so far have shown that children with ADHD have increased body weight or BMI in comparison with pediatric norms or control groups [6,13,20,36]. Holtkamp et al. found that boys with ADHD had higher BMI and were more often overweight or obese compared to the general population [10]. Among children and adolescents, BMI changes dynamically according to age and height, and it is therefore necessary to use appropriate centiles [37]. Not all studies agree, but from the presented outcomes, we would assume that children with ADHD are overweight because they consume sweetened drinks more often. A large consumption of fermentable carbohydrates (e.g., lemonades and fruit juices) presents a high sugar content with quite low pH-values, and might be an essential risk factor for overweight and obesity [38]. It is worth noting that the relationship between waist circumference and high intake of sweetened beverages in our study was found close to significance in children with ADHD.

However, the adverse health effects of obesity might result from other dietary habits, including sugar-sweetened beverages but also a nutritional profile focused on fatty or processed food products. In many studies, discrepancies have appeared, indicating that there is no simple relationship between ADHD and obesity. It can be assumed that unspecified intermediaries have a role in the observed incidence of obesity in children with ADHD. The ADHD-obesity relationship can be explained by genes [9,12], neurobiological features [37], deficits in executive functions [39], fetal programming [36], sleep disorders (circadian rhythm) and incorrect eating habits [23], high-fat diet [22], reduced physical activity [20,21], and stress [40], as well as a sedentary lifestyle. It is also suggested that the association of ADHD with obesity may be a side effect of adaptation to a poor food environment [41], but few studies have tested this hypothesis and the results are inconclusive [1].

ADHD is a significant factor in obesity and excess weight, disregarding pharmacological treatment, socio-economic status, and most concomitant disorders connected with ADHD [21]. A possible determining factor that predisposes children with ADHD to an excess of weight is the inability to cope with difficult situations and experienced emotions. Instant gratification in the form of highly calorific food is often a natural and available way of compensating for difficulties in school, social failures, or family conflicts [41,42].

The relationship between clinical and biological factors in ADHD has been considered along with oral health, but this is the first study based on ICDAS evaluation among ADHD children.

To the best of our knowledge, previous studies used only dmft/DMFT systems for caries analysis, and there are conflicting reports according to dental status [24,25,26,27,28]. In the course of our literature review, we detected a lack of dental investigations assembling ICDAS compiled with anthropometric information collected from children with ADHD. The ICDAS method has several benefits, including a high accuracy, since coding the varied lesions helps clinicians and researchers to differentiate the diverse stages of the disease [42]. Based on the ICDAS results, it could be assumed that children affected by ADHD present a higher risk of dental caries in both primary and permanent teeth. However, epidemiological surveys performed in the general population give conflicting outcomes regarding the relationships between BMI and the frequency of dental caries. In the available literature, we could find the opinion that obese children do not have extensive tooth decay, while other studies reject this hypothesis [38,43,44,45].

So far, we have been missing oral health assessment among the Polish subpopulation with ADHD, particularly with the use of ICDAS scoring. Unfortunately, previously published results were limited, and attention has been paid to the overall frequency of caries in children with ADHD [25,26,46,47,48]. We could only find dental examinations performed with the WHO-DMFT caries index (determining the prevalence of decayed, missing and filled teeth, dmft designed for primary dentition, DMFT for secondary dentition). In a Scandinavian study based on a population with ADHD, the mean DMFT was 2.8 ± 4 in comparison to children not suffering from ADHD, where the mean DMFT was 2.2 ± 3.2 [25]. From other studies, the obtained DMFT results varied according to the occurrence of caries; however, DMFT was never scored below 2 according to permanent teeth [46,47,48,49]. In previous studies, subjects differed in age from the present study (average age was 13 years old), and there was no data to compare the condition of primary dentition. Moreover, most studies were performed in a small number of children with ADHD and consisted of 20 to 30 participants [25,26]. We found only one similar study, where the number of participants was near 50 ADHD children, but again the average age was estimated above 12 years old [46,47,48,49].

Based on the results of a national survey conducted in Poland on children’s oral health among healthy schoolchildren aged 5−6 and 12 years old [50], it should be noted that the data would be difficult to compare due to wide community and age ranges. In comparison to the national data, the number of carious teeth in the age group of children aged 5–7 years is still high [50,51]. The national report also indicates that younger and younger children have dentition affected by tooth decay as early childhood caries (ECC). Recently performed national monitoring research among children revealed dental caries in 76.9% of 5-year-old children and 89.4% of 7-year-old children [51]. Even in Germany, with a well-established healthcare system, 13.7% of 3-year-olds, 43.6% of 6–7-year-olds, and 21.2% of 12-year-old children had at least one tooth with a caries experience [52]. Other developed countries like Australia and the USA show a comparable high prevalence of ECC [53,54].

The present study revealed the frequent consumption of sugars as one of the critical factors for the intensive development of caries disease in children with ADHD. However, we have to remember that children with ADHD are difficult dental patients, and their parents have significant problems in maintaining their proper oral hygiene. It is possible that a lack of oral hygiene and poor cooperation in the dental office might also be reasons for the development of caries, and not just the consumption of sugars. Our opinion is based on a recruitment period of the study where a dental examination was ultimately performed in thirty-nine children among fifty-four qualified ADHD children. Therefore, in the case of ADHD diagnosis, early prevention programs against dental caries and overweight with limited sweetened drinks consumption might be considered. The obtained relationship between sugar intake and dental caries among children with ADHD suggests further research is necessary, including larger groups of ADHD patients.

Our data differ slightly from data reported by previous research. This could be because of the age of the study group as well as the different nature of the project. Our investigation was based on the voluntary participation of parents and ADHD/healthy children, an original selection of employed diagnostic scales for ADHD, a smaller study group, and a narrower study population (one urban area and its surroundings). The present study was also limited by its cross-sectional design and general measures of nutritional status. Laboratory instruments for body composition measurements are more accurate, but difficulties (e.g., in transportation) limit their application. All mentioned differences do not, however, undermine the representativeness of the groups we examined.

## 5. Conclusions

Our main conclusion is that the outcomes of the present survey support the hypothesis that ADHD might be related to excessive weight gain. Unhealthy dietary habits related to ADHD may lead to an increased rate of overweight in this group of patients, but may also affect oral health. Children with diagnosed ADHD may present a higher risk of dental caries in primary and permanent dentition than the healthy population. It can be assumed that tooth decay is associated with the consumption of sugar-sweetened drinks, and that this is one of the essential factors for the intensive development of caries disease in children with ADHD.

## Figures and Tables

**Table 1:** Inclusion and exclusion criteria for both groups.

Criteria for Inclusion into the Study Group	Criteria for Inclusion into the Control Group	Criteria for Exclusion from Study and Control Groups
Children of both sexes aged 6–11	Children of both sexes aged 6–11	Children with disorders of central nervous system (e.g., epilepsy, serious injuries, and CNS infections)
Children with diagnosed ADHD in accordance with ICD-10 and DSM-V diagnostic criteria (diagnosis confirmed by two independent psychiatrists based on a standardized and structured interview)	Lack of mental disorders—assessment with the use of MINI-Kid questionnaire [30]	Co-existing: schizophrenia, bipolar affective disorder, any serious somatic disorders
Clinically significant ADHD symptoms lasting over 6 months	Parent or legal guardian approval	Chronic somatic diseases
Children without hereditary mental disorders (first-degree relatives)		Persistent pharmacotherapy, hormonotherapy
Parent or legal guardian approval		Lack of acceptance from parents or legal guardians

ADHD—Attention Deficit Hyperactivity Disorder; ICD-10—International Statistical Classification of Diseases and Related Health Problems (10th edition), DSM-IV—Diagnostic and statistical manual of mental disorders (4th ed.), DSM-V—Diagnostic and statistical manual of mental disorders (5th ed.), MINI-Kid—MINI International Neuropsychiatric Interview for Kids; CNS—Central Nervous System.

**Table 2:** Selected statistics of developmental parameters for ADHD and control groups.

Group Variables	ADHD *n* = 39	Control *n* = 80	*p*-Value
Age (years)	8.8 ± 1.2 * 8 (6–11)	8.0 ± 1.1 * 8 (6–10)	ns
Height (cm)	135.6 ± 7.6 * 135 (122–157)	132.9 ± 8.5 * 133 (115–156)	ns
Body weight (kg)	33.6 ± 10.3 * 31.8 (16.3–75.8)	29.3 ± 6.4 * 27.9 (19.3–52.6)	0.008 **
BMI (kg/m^2^)	18.1 ± 4.1 * 16.7 (10.9–30.7)	16.3 ± 2.1 * 16 (12.8–23.4)	0.016 **
Waist size (cm)	63.3 ± 11.5 * 60 (49–101)	59.9 ± 6.5 * 59 (38–80)	ns
Hip size (cm)	75.6 ± 9.1 * 74 (57–104)	70.3 ± 6.1 * 70 (50–88)	0.001 **

* Mean ± standard deviation, **Median** (min–max), ** *p*-value with statistical difference, *n*—number of examined children, ns—not significant value in statistical analysis.

**Table 3:** Statistical analysis for primary dentition according to ICDAS score in ADHD and control groups.

Group Parameter	ADHD *n* = 39	Control *n* = 80	*p*-Value
Number of primary teeth	11.2 ± 3.6 *	12.3 ± 2.4 *	ns
ICDAS = 0	4.2 ± 3.4 * 3 (0–13)	9.3 ± 3.5 * 9 (3–20)	<0.001 **
ICDAS = 1	2.1 ± 1.8 * 2 (0–10)	0.5 ± 0,9 * 0 (0–5)	<0.001 **
ICDAS = 2	1.4 ± 1.2 * 2 (0–4)	1.0 ± 1.1 * 1 (0–5)	0.094 **
ICDAS = 3	0.2 ± 0.5 * 0 (0–2)	0.5 ± 1.0 * 0 (0–5)	ns
ICDAS = 4	0.4 ± 1.2 * 0 (0–7)	0.4 ± 0.8 * 0 (0–4)	ns
ICDAS = 5	1.7 ± 2,2 * 1 (0–10)	0.3 ± 0.8 * 0 (0–3)	<0.001 **
ICDAS = 6	1.1 ± 2.0 * 0 (0–10)	0.2 ± 0.6 * 0 (0–2)	0.009 **

* Mean ± standard deviation, **Median** (min–max), ** *p*-value with statistical difference. *n*—number of examined children, ns—not significant value in statistical analysis.

**Table 4:** Statistical analysis for permanent dentition according to ICDAS score in ADHD and control groups.

Group Parameter	ADHD *n* = 39	Control *n* = 80	*p*-Value
Number of permanent teeth	12.5 ± 3.5 *	11.2 ± 2.9 *	ns
ICDAS = 0	10.3 ± 3.9 * 10 (0–20)	10.1 ± 2.8 * 10.5 (0–14)	ns
ICDAS = 1	0.9 ± 1.1 * 1 (0–4)	0.6 ± 0.9 * 0 (0–3)	0.071 **
ICDAS = 2	0.6 ± 1.0 * 0 (0–3)	0.5 ± 0.9 * 0 (0–3.0)	ns
ICDAS = 3	0.6 ± 0.9 * 0 (0–4)	0.1 ± 0.3 * 0 (0–2)	0.011 **
ICDAS = 4	0.1 ± 0.3 * 0 (0–1)	0.01 ± 0.1 * 0 (0–1)	ns
ICDAS = 5	0.03 ± 0.2 * 0 (0–1,0)	0.01 ± 0.1 * 0 (0–1.0)	ns
ICDAS = 6	0.03 ± 0.2 * 0 (0–1.0)	none	-

* Mean ± standard deviation, **Median** (min–max), ** *p*-value with significant difference. *n*—number of examined children, ns—not significant value in statistical analysis.

**Table 5:** Statistical analysis of questionnaire results related to food/drink-related questions (%).

Positive Answers (%)	Groups		
	ADHD	Control	*p*-Value
	*n* = 39	*n* = 80	
Sugar confectionery consumption every day (%)	94.9	38.9	0.001 **
Sugar snacks consumption between main meals (%)	84.6	28.8	0.001 **
Sugar snacks consumption controlled by the parents (%)	30.8	70	<0.001 **
Drinking of natural water (%)	28.2	50	0.024 **
Drinking of carbonated sweet drinks (%)	69.2	23.8	0.001 **
Drinking of sweet beverages between main meals (%)	100	73.8	<0.001 **

** *p*-value with significant difference, *n*—total number of subjects.

## Abbreviations

ADHD—Attention Deficit Hyperactivity Disorder, BMI—Body Mass Index, DSM-V—Diagnostic and Statistical Manual of Mental Disorders (5th ed.), ECC—Early Childhood Caries, GCP—Good Clinical Practice, ICD-10—International Statistical Classification of Diseases and Related Health Problems (10th ed.), ICDAS II—International Caries Detection and Assessment System, second edition, WHO—World Health Organization.
